# Implementing a Medicines at Transitions Intervention (MaTI) for patients with heart failure: a process evaluation of the Improving the Safety and Continuity Of Medicines management at Transitions of care (ISCOMAT) cluster randomised controlled trial

**DOI:** 10.1186/s12913-024-11487-x

**Published:** 2024-10-09

**Authors:** Catherine Powell, Hanif Ismail, Liz Breen, Beth Fylan, Sarah L Alderson, Chris P Gale, Peter Gardner, Jonathan Silcock, Bonnie Cundill, Amanda Farrin, Ellen Mason, Lauren Moreau, David P Alldred, ISCOMAT Programme Management Team, Catherine Powell, Hanif Ismail, Liz Breen, Beth Fylan, Sarah L Alderson, Chris P Gale, Peter Gardner, Jonathan Silcock, Bonnie Cundill, Amanda Farrin, Ellen Mason, Lauren Moreau, David P Alldred, Gerry Armitage, Alison Blenkinsopp, Robert Turner, Andrew Taylor, Ian Kellar, Suzanne Hartley, Chris Bojke, John Wright

**Affiliations:** 1 School of Pharmacy and Medical Sciences, University of Bradford, Bradford, UK; 2 Wolfson Centre for Applied Health Research, Bradford, UK; 3 Research and Innovation Centre, Leeds Teaching Hospitals NHS Trust, Leeds, UK; 4 NIHR Yorkshire and Humber Patient Safety Translational Research Centre, Bradford Institute for Health Research, Bradford, UK; 5 Leeds Institute of Health Sciences, University of Leeds, Leeds, UK; 6 Department of Cardiology, Leeds Teaching Hospitals NHS Trust, Leeds, UK; 7 Leeds Institute for Data Analytics, University of Leeds, Leeds, UK; 8 Leeds Institute of Cardiovascular and Metabolic Medicine, University of Leeds, Leeds, UK; 9 Clinical Trials Research Unit, Leeds Institute for Clinical Trials Research, University of Leeds, Leeds, UK; 10 School of Healthcare, University of Leeds, Leeds, UK

**Keywords:** Implementation, Qualitative methods, Medicines management, Heart failure

## Abstract

**Background:**

Heart failure is a major global health challenge incurring a high rate of mortality, morbidity and hospitalisation. Effective medicines management at the time of hospital discharge into the community could reduce poor outcomes for people with heart failure. Within the Improving the Safety and Continuity Of Medicines management at Transitions of care (ISCOMAT) programme, the Medicines at Transitions Intervention (MaTI) was co-designed to improve such transitions, with a cluster randomised controlled trial to test effectiveness. The MaTI includes a patient toolkit and transfer of discharge medicines information to community pharmacy. This paper aims to determine the degree to which the intervention was delivered, and identify barriers and facilitators experienced by staff for the successful implementation of the intervention.

**Methods:**

The study was conducted in six purposively selected intervention sites. A mixed-methods design was employed using hospital staff interviews, structured and unstructured ward observations, and routine trial data about adherence to the MaTI. A parallel mixed analysis was applied. Qualitative data were analysed thematically using the Framework method. Data were synthesised, triangulated and mapped to the Consolidated Framework for Implementation Research (CFIR).

**Results:**

With limited routines of communication between ward staff and community pharmacy, hospital staff found implementing community pharmacy-related steps of the intervention challenging. Staff time was depleted by attempts to bridge system barriers, sometimes leading to steps not being delivered. Whilst the introduction of the patient toolkit was often completed and valued as important patient education and a helpful way to explain medicines, the medicines discharge log within it was not, as this was seen as a duplication of existing systems. Within the CFIR the most applicable constructs were identified as ‘intervention complexity’ and ‘cosmopolitanism’ based on how well hospitals were networked with community pharmacies, and the availability of hospital resources to facilitate this.

**Conclusion:**

The MaTI was generally successfully implemented, particularly the introduction of the toolkit. However, implementation involving community pharmacy was more challenging and more effective communication systems are needed to support wider implementation.

**Trial registration:**

11/04/2018 ISRCTN66212970. https://www.isrctn.com/ISRCTN66212970.

**Supplementary Information:**

The online version contains supplementary material available at 10.1186/s12913-024-11487-x.

## Contributions to the literature

- Enhances our understanding of the key barriers and facilitators experienced by staff that may be present in implementing a complex intervention across a transition involving hospital and community pharmacy
- Illustrates the application of the CFIR in a mixed method process evaluation using interviews with patients and hospital staff, and observations

## Background

Twenty-six million people globally live with heart failure, with 900,000 people affected in the United Kingdom and numbers rising [1]. Heart failure can be managed through pharmacological treatments, such as angiotensin-converting enzyme (ACE) inhibitors, beta adrenoceptor antagonists and diuretics [2]. When medicines are effectively optimised, rates of hospitalisation decrease; and quality of life and mortality rates improve [3]. However, achieving optimisation can be challenging when patients living with heart failure frequently transition between hospital and home, and readmission rates can be as high as 50% [4]. A key issue in transitions is the poor communication of treatment between health care professionals [5]. Therefore, creating effective communication systems when patients with heart failure are discharged from hospital is essential.

The Improving the Safety and Continuity Of Medicines management at Transitions of care (ISCOMAT) programme aimed to improve the use of prescribed medicines when patients with heart failure are discharged from hospital. A Medicines at Transitions Intervention (MaTI) was co-designed with healthcare professionals and patients, and consisted of a patient held ‘My Medicines Toolkit’ in booklet format. The toolkit included: (1) My Healthcare Team, with contact details of their healthcare team; (2) My Medicines Checklist to help manage medicines; (3) Managing My Medicines, with information about the patient’s medicines, side effects and how to take them; (4) Managing my Symptoms, ‘traffic lights’ to help patients monitor changes to their symptoms of worsening heart failure and know when they should seek help; and a pull-out sheet for hospital staff to complete medicines information and enabling patients to monitor their condition.

**Fig. 1:**
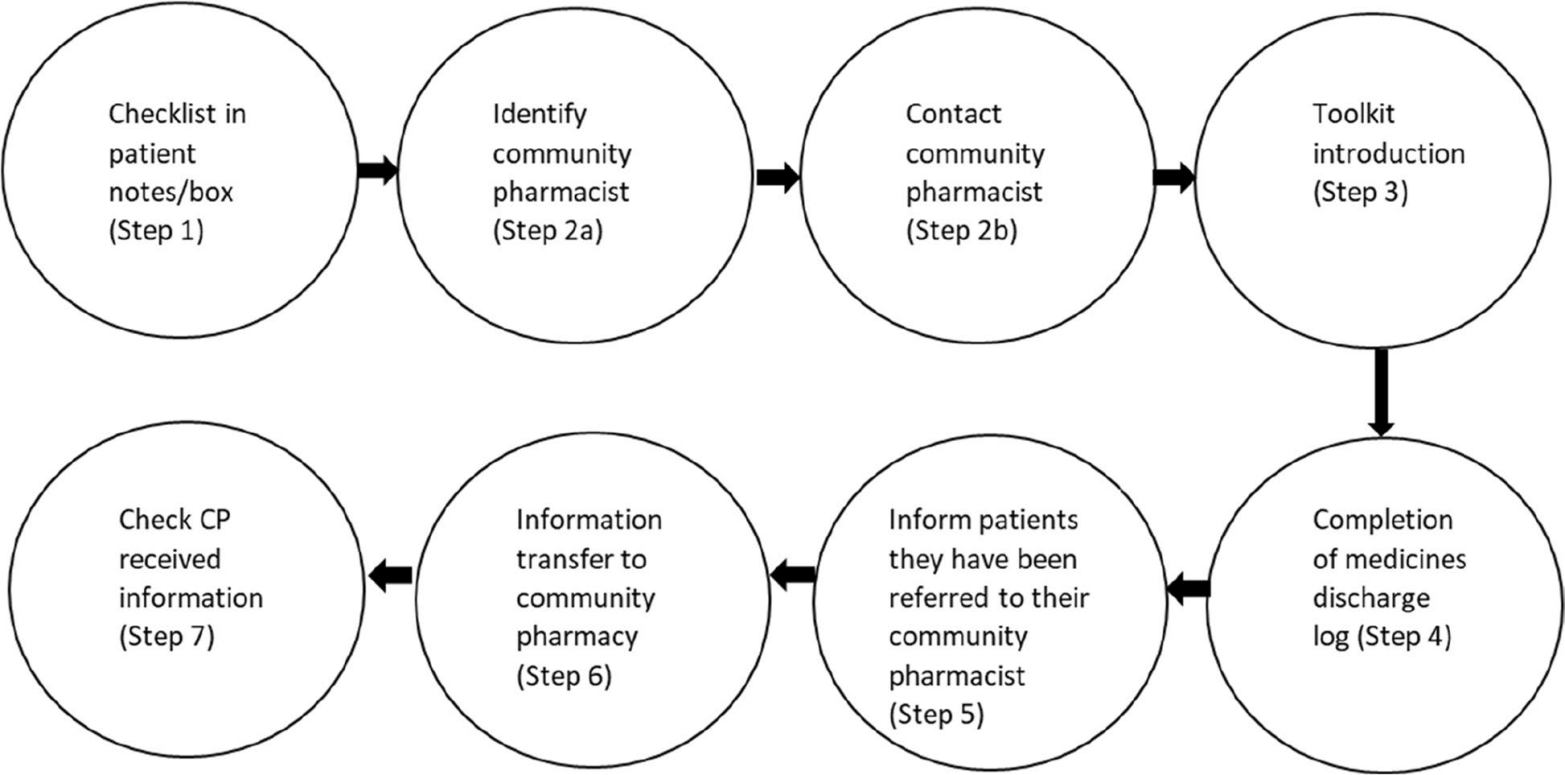
Medicines at Transitions Intervention (MaTI) 7 steps

The discharge medicines list was transferred (by post, facsimile, or electronically depending on site preference) by the hospital to the community pharmacy to facilitate medicines reconciliation. Community pharmacists were encouraged to invite patients for a medicines discussion or Medicines Use Review (MUR) [6]. Hospital staff were provided with face-to-face and online training and supporting materials, including an implementation guide and a script to introduce the toolkit to patients. Figure 1 indicates how MaTI is delivered through 7 steps by hospital staff.

Following feasibility testing, [7] intervention effectiveness was assessed in a cluster randomised controlled trial (cRCT) in NHS trusts in England over 12 months, with a recruitment target of 50 patients per site (2100 patients total) [8]. 44 clusters were randomised, of which 43 opened to recruitment. Alongside the trial, a process evaluation was conducted. To identify implementation determinants we selected the Consolidated Framework for Implementation Research (CFIR) as an appropriate framework to guide our analysis during and post-implementation [9]. We have previously published patient experiences of the MaTI. This paper focuses on intervention implementation by hospital staff [10].

## Methods

The key objectives were to:

- Determine the degree to which the intervention was delivered
- Identify barriers and facilitators experienced by staff for the successful implementation of the intervention

The study design was a parallel mixed synthesis study using quantitative and qualitative data from six intervention sites of the total 43 recruiting clusters in the cRCT. We have previously published the protocol [11]. Methods involved non-participant observations, semi-structured interviews and analysing trial fidelity data on adherence to MaTI. We intended to interview hospital staff involved in the intervention, such as nurses, pharmacists, pharmacy technicians, and site coordinators (research nurses at each site), as well as community pharmacists, and community heart failure nurses [11]. However due to the health and social implications of the COVID-19 pandemic and the prioritisation of COVID-19 research [12], we were required to adapt our approach.

Our Patient Led Steering Group has been involved throughout the ISCOMAT programme on key aspects such as facilitating the intervention co-design process and co-analysing patient interviews. For further information on the Patient Led Steering group see Powell et al. (2021) [13]. The trial and process evaluation received approval by Research Ethics Committee and the UK Health Research Authority REC: 18/YH/0017/IRAS: 231 431. This study is reported according to the consolidated criteria for reporting qualitative research [14] (See Additional File 1).

### Sampling and recruitment

We purposively sampled six intervention sites using three criteria: university and non-university hospitals, the method for transferring medicines discharge information to community pharmacists (e.g., an electronic system such as PharmOutcomes^®^ [15]), and geographical location (see Additional file 2 for site characteristics). Explanations of differences between sites have been provided to effectively apply the CFIR, revealing differences in barriers and facilitators to implementing the intervention.

Permission was sought from each site to conduct non-participant ward-level observations, and staff were provided with information sheets and could opt out if they wished. A script for informing patients of the researchers’ presence was supplied. Interviewees were identified during ward observations and from site coordinators. Informed consent was obtained from all the participants. Procedures for recruiting patients to the cRCT are outlined in the trial protocol [8].

### Data collection

Once sites had implemented the intervention for at least 6 months, observations of clinical staff and interactions with patients were conducted over 2.5 hours at each process evaluation site by female (CP) and male (HI) researchers. The observations focused on the discharge process, introduction of the My Medicines Toolkit (the Toolkit), and ward culture. Observations were structured, focusing on the intervention delivery; and unstructured, focusing on ward culture (see Additional files 3 and 4 for data collection tools). Community pharmacy data were sought from pharmacies through surveys (see Additional file 5).

Once trial recruitment was complete in sites, semi-structured interviews with hospital staff were conducted using an interview schedule by experienced qualitative researcher HI. The schedule was informed by the CFIR, covering staff experiences in delivering the intervention (see Additional file 6). Interviews lasting approximately 45 minutes were audio recorded and transcribed verbatim. The timing of interviews was designed to ensure we did not influence implementation during the trial. In Site 2, interviews were conducted 2 weeks post-trial recruitment as planned. In the other sites, interviews were conducted several months later in August/September 2020 after all sites had closed to trial recruitment due to COVID-19.

### Data analysis

Data for this process evaluation were analysed prior to main trial analysis to reduce bias in our interpretation. A two-stage approach to qualitative analysis was conducted by two researchers. First, Framework analysis was applied to interviews and unstructured observations, identifying barriers and facilitators to implementation [16, 17].

Framework analysis involved 7 key stages. In stage 1 audio recordings of interviews were transcribed verbatim by a transcription company. Stage 2 involved familiarisation with data, where all data were read, and relevant notes made in the margins. In stage 3 data were coded applying an inductive approach. Stage 4 involved developing a working analytical framework, where CP and HI agreed on codes for subsequent data collection. In stage 5 CP and HI organised data into the analytical framework. Stage 6 involved charting data into the framework matrix. Summaries were created from data and charted onto the CFIR by site and intervention steps [9]. We applied the competing values framework to understand the ‘culture’ domain within sites [18, 19]. We added additional constructs of person-centred care and safety which were considered appropriate for a medicines management intervention in a hospital setting. The competing values framework was applied to collated observation and interview data. Decisions on the appropriate constructs were agreed by researchers CP and HI. In stage 7 data were interpreted.

Patient interviews were co-analysed with researchers CP, HI and the ISCOMAT patient led steering group. Qualitative staff data were analysed by CP and HI. The thematic analysis was iterative with regular discussions taking place with the process evaluation team.

All qualitative analysis was conducted using NVivo 12 [20]. A project template with CFIR construct and domain names were directly imported into NVivo via the CFIR website [21].

Quantitative data including structured observations were descriptively analysed (see Additional file 7). Additional data from the wider trial were triangulated with process evaluation data to inform, contextualise, and explain findings. These included a Site Feasibility Questionnaire (questions to assess sites eligibility), MaTI checklist completed in the hospital (monitors adherence to intervention components), and a community pharmacy data collection form (assessed implementation within community pharmacy).

A parallel mixed analysis was applied to qualitative and quantitative data, with both independently analysed, and integrated using meta-inferences [22]. Data were integrated through applying the CFIR. All CFIR constructs and domains were considered in the analysis.

## Results

Structured and unstructured observations of up to 2.5 hours with breaks, were conducted separately, each by two researchers. Unstructured observations were conducted in six sites, and one structured observation was conducted by two researchers in five of the sites. Eleven staff interviewees were recruited, with no dropouts. Table 1 indicates the types of staff recruited by site. Some CFIR constructs were not relevant or had little evidence to support their relevance. Table 2 shows the domains and constructs that were relevant in explaining barriers and facilitators to implementation.

**Table 1:** Hospital staff interviews

Site	Staff role
Site 1	Site co-ordinator (research nurse organising intervention delivery) Ward Pharmacist
Site 2	Site co-ordinator Ward Pharmacy Technician Ward nurse 1 Ward nurse 2
Site 3	Senior ward Pharmacist Ward Pharmacist
Site 4	Site co-ordinator Heart Failure Specialist Nurse
Site 5	None
Site 6	Site co-ordinator

**Fig. 2:**
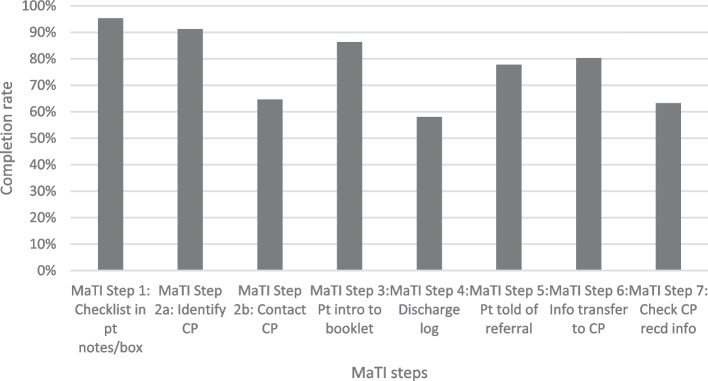
All intervention sites fidelity to MaTI (of 593 forms received)

**Fig. 3:**
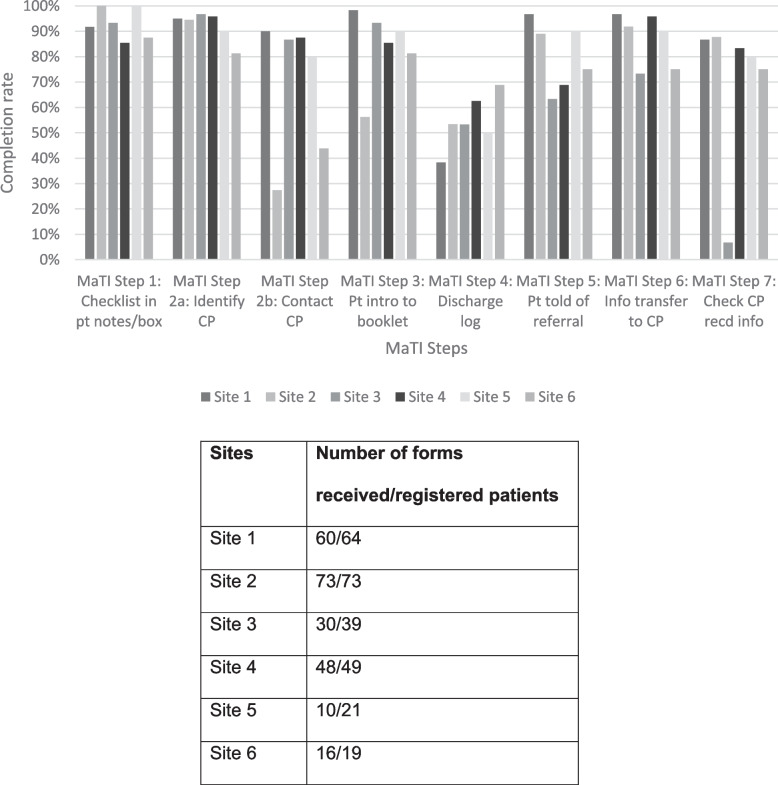
Process evaluation sites fidelity to MaTI (of forms received)

The figures below indicate the degree to which the intervention was delivered. Figures 2 and 3 illustrate the extent to which each step was completed per returned MaTI checklist form for each patient. Figure 2 outlines fidelity across all intervention sites, and Fig. 3 indicates fidelity across process evaluation sites. Qualitative analysis on completion of the MaTI is outlined in ‘Process, executing’ domain of the CFIR analysis.

Routine trial data indicated that in all sites, checklist in patients notes/box (Step 1), identify community pharmacist (Step 2a) and toolkit introduction (Step 3) were most frequently implemented. This was reflected in the six process evaluation sites, apart from the toolkit introduction (Step 3) in Site 2, with relatively low intervention fidelity (65.2%). Completion of medicines discharge log (Step 4) was the least completed step across all sites. In the process evaluation sites, Site 1 completion of medicines discharge log (Step 4) was completed for only 38.3% of patients. Contact community pharmacist (Step 2b) had relatively low completion overall (64.6%), and this was reflected in process evaluation sites Site 2 (38.3%) and Site 6 (27.4%). However, relatively high completion of contact community pharmacist (Step 2b) was evidenced across process evaluation sites Site 5 (80%), Site 4 (87.5%), Site 3 (86.7%) and Site 1 (90.0%). Check community pharmacist received information (Step 7) in Site 3 was an outlier with only 6.7% completion, relatively low when compared with all sites (63.2%).

Hospital staff reported information transfer to community pharmacy (Step 6) had a relatively high completion rate of 68% across all sites, and at least 73% competition in process evaluation sites, however community pharmacy survey data suggested otherwise.

Across all sites, 124 community pharmacy surveys were returned. 75/124 (60.4%) community pharmacists reported not receiving discharge information, and data were missing for 12/124 (9.6%). Of those 75, the information transfer to community pharmacy (Step 6) was reported as completed by hospital staff for 71 (94.6%) patients.

Across process evaluation sites, 75 community pharmacy surveys were returned (Site 1:24, Site 2:30, Site 3:6, Site 4:8, Site 5:2, Site 6:5). Of those, the information transfer to community pharmacy (Step 6) was completed for 64/75 (Site 1:24, Site 2:29, Site 3:4, Site 4:8, Site 5:2, Site 6:5). However, only 22/75 community pharmacists reported receiving information from the hospital, with 46 community pharmacists reporting they did not receive it, and 7 forms had missing data.

Table 2 presents barriers and facilitators to the intervention implementation by CFIR domain and constructs. Data were drawn from staff interviews, and structured and unstructured observations. These provided insight into the routine trial data.

**Table 2:** Barriers and facilitators to the intervention implementation by Consolidated Framework Implementation Research domains and constructs. (Adapted from CFIR codebook) [21]

CFIR domains and constructs	Barriers and facilitators
**Innovation characteristics**	
Adaptability	Facilitator: MaTI was adapted to reflect differing information transfer systems, staff roles, family and patient involvement, and ways of working.
Complexity	Barrier: Completion of medicine discharge log (Step 4) was complex in finding the correct time to complete it.
Design quality and packaging	Facilitator: The patient toolkit was felt to have benefited staff and patients. Helped staff discuss medicines with patients. The structure, colour and style were appealing.
**Outer setting**	
Patient needs and resources	Facilitator: Some staff were motivated to deliver intervention components when they felt it addressed patient needs. Patient education was regarded as important for supporting patients to continue monitoring and understanding their medicines.
External policy and incentives	Facilitator: Pharmacist practice and education, supported the delivery of the intervention in some sites.
Cosmopolitism	Barrier: Hospitals not well networked with community pharmacy. Lack of receptiveness of community pharmacy to the intervention in relation to contact community pharmacist (Step 2b), information transfer to community pharmacy (Step 6) and check community pharmacist received information (Step 7).
**Inner setting**	
Networks & Communications	Facilitator: Existing communication systems and relationships supported implementation of the intervention steps which were delivered over a lengthy period of time.
Culture	Facilitator: Person-centred care, safety, clan, and hierarchical culture.
Implementation Climate	Facilitator: The intervention was compatible with existing practice as it was close to the norm. Barrier: Channels of communication between hospital and pharmacy unreliable. Relative priority of MaTI intervention reduced given competing sources.
Readiness for Implementation	Barrier: Two of the lesser implemented steps of MaTI, contact community pharmacist (Step 2b) and completion of medicines discharge log (Step 4), were impacted by resource deficiencies.
**Characteristics of Individuals**	
Knowledge & Beliefs about the Innovation	Barrier: Staff reported some patients felt the intervention was already being carried out in community pharmacy. Some ward staff lacked enthusiasm for the intervention.
Other Personal Attributes	Facilitator: Site coordinator ability to increase staff confidence and sense of self-efficacy in delivering MaTI.
**Process**	
Planning	Facilitator: Developing a Standard Operating Procedure to implement transfer of patient information to community pharmacy in one site.
Engaging	Barrier: Patient engagement not encouraged as much in some sites e.g., asking questions. Facilitator: Pharmacist highly engaged in delivering intervention in one site. Training cited as important in some sites.
Executing	Barrier: Completion of medicines discharge log (Step 4) had poor implementation across sites.

**Table:** 

Barriers	Facilitators
• Complexity • Cosmopolitism • Implementation Climate • Readiness for Implementation • Knowledge & Beliefs about the Innovation • Engaging • Executing	• Adaptability • Design quality and packaging • Patient needs and resources • External policy and incentives • Networks & Communications • Culture • Implementation Climate • Other Personal Attributes • Planning • Engaging

### Intervention characteristics domain

Each domain was defined as ‘distinguishing’ (influence differentiation in implementation), or not. Intervention characteristics was not a highly distinguishing domain, with adaptability and intervention design having positive impacts on implementation, and intervention complexity presenting limitations to implementation. Data sources ‘interviews’, ‘observations’ and ‘surveys’ are outlined as subheadings below.

### Facilitators

#### Interview data

Adaptability was an important construct, with MaTI adaptable across all sites. MaTI was adapted to differing information transfer systems, staff roles, family and patient involvement, and ethos.

Site 1 trained ward staff to use PharmOutcomes^®^ to transfer of discharge medicines information, [15] whereas in Site 3, pharmacists used PharmOutcomes^®^, [15] perhaps reflecting their approach of pharmacists driving the intervention in Site 3.

*“We just sort of told [the ward staff] how to get into* [PharmOutcomes^®^ [15]] *set the patient up on there*,* and then how to just transfer information*,* with a user guide.”* Site 1 Ward pharmacist.

The design and quality of the toolkit was well regarded across all sites. It was felt to have benefited staff and patients. Staff found it helped them discuss medicines with patients. The presentation was thought to be very appealing in structure, colour and style,

“*it was more glossier and more colourful version of the booklets they used to have…they found that useful yes to explain…very appreciative of the amount of information in it and how easy it is for the patients.”* Site 2 Coordinator.

#### Barriers

The complexity of the intervention was important for implementation across most sites. Completion of the medicines discharge log (Step 4) could be challenging where last-minute medicine changes were made (Site 6), or during out-of-hours when coordinators were not available to support staff (Site 1). Site 2 provided the discharge summary in the back of the toolkit as a work around to completing Step 4, given the lack of time at discharge.

*“we didn’t fill them out if I’m honest*,* we didn’t just because it was time-consuming; that was the only thing. But what we did do is we put the discharge summary in the back.”* Site 2 Cardio nurse.

### Outer setting domain

The outer setting was a distinguishing construct, despite limited mention of external factors having an influence on implementation; with some sites demonstrating prioritisation of patient need and adopting differing approaches to toolkit delivery in relation to such needs.

### Facilitators

#### Interview data

Staff were motivated to deliver intervention components when they felt they addressed patient needs. This domain was also relevant to compatibility as staff held values and beliefs which facilitated implementation. Patient education was regarded as important for supporting patients to continue monitoring and understanding their medicines.

*“There’s such a high rate of patients coming back into hospital*,* so this is really important to educate patients so they can monitor themselves at home.”* Site 2 Ward nurse.

Staff adapted toolkit delivery according to perceived patient engagement and knowledge of heart failure.

*“Somebody who is proactive*,* it is then beneficial to them…Patients who just said they didn’t want to take part…we still left information for them*,* it’s their choice.”* Site 1 coordinator.

Some evidence suggested changes in pharmacist practice and education supported intervention delivery with pharmacists expected to have a greater role discussing medicines with patients.

### Barriers

Cosmopolitism (how well networked the organisation is), was an important construct. Communication between hospital and community pharmacy posed significant challenges. Contact community pharmacist (Step 2b), information transfer to community pharmacy (Step 6) and check community pharmacy received information (Step 7) were challenging to implement.

### Survey data

Community pharmacies were not prepared to receive information from hospitals and lacked knowledge of ISCOMAT. Limited community pharmacy data indicated some had difficulty understanding ‘medicines reconciliation’. Occasionally only locum and relief pharmacists were available, thus staff who originally received the study letter were not available.

### Interview data

Sites sometimes needed to spend significant time and resource to complete these steps.

*“It was more trying…to get them* [community pharmacy] *confirmation that they’d received it* [Discharge medicines information].*”* Site 4 Site coordinator.

*“The pharmacy…didn’t have an NHS.net account*,* so they couldn’t send patient identifiable data across*,* so that was the biggest stumbling block and on some occasions we even just hand delivered it…so that we knew it had gone securely.”* Site 4, Heart failure specialist nurse.

*“Just getting*,* trying to get hold of the one* [pharmacist] *that was on shift when the letter arrived*,* and getting them to remember…what part of the letter we spoke about.”* Site 6.

Community pharmacists were deemed to be more responsive if communicating with a hospital pharmacist.

*“Coming from a pharmacist to a pharmacist*,* I think they would probably acknowledge things a little bit better and a bit quicker…There were only a couple of pharmacies that would reply and go*,* “Yes*,* got it.”* Site 4.

Site 3 reported fewer communication issues with community pharmacy using their existing PharmOutcomes ^®^ [15] systems via hospital pharmacists; however, despite having this in place, they only checked community pharmacists received information (Step 7) for 6.7% of patients.

### Inner setting domain

The inner setting was not a highly distinguishing construct. However, some sites were more able to readily implement the intervention according to staff role and availability. All constructs had a strong influence on implementation.

### Facilitators

Networking and communication had a strong positive influence on implementation at all sites (excluding Site 5 where no data were available).

#### Interview data

The intervention steps were delivered over a lengthy period, which required sites to develop effective network and communication systems to work around staff availability. The extract below illustrates how some steps of the intervention were organised between different staff.

*“The heart failure team…basically said to all the other nursing staff or doctors on the ward ‘Let us know if you see a heart failure patient but we’ll kind of take over…that was the most efficient… isn’t a process where you do it all at once*,* you’re kind of identifying*,* giving the information.”* Site 3 Hospital pharmacist.

The implementation climate had a positive influence on implementation across Sites 6, 2, 1, and 3. The implementation climate had a negative impact in Site 4, and to some extent in Site 5 based on limited data. Compatibility and relative priority of the intervention were key factors influencing implementation.

### Barriers

Other intervention steps were less distinguishing in terms of compatibility, with sites describing providing patient education as the norm, and the toolkit enhanced this to some extent.

*“For the majority of…* [patients], *it…reinforced the discussions that we normally have at discharge*,* about their medication*,* but probably it was definitely more in depth.”* Site 6 Site coordinator.

The lack of available resources was a hinderance across most sites. Having the appropriate staff available on the ward to deliver the intervention could enhance implementation for different phases of MaTI. Two of the lesser implemented steps of MaTI, communication with community pharmacy and completion of the medicines discharge log (Step 4), were impacted by this.

Leadership engagement had some positive influence in Site 2, but this was offset by available resources which led to a strong negative influence on implementation. The intervention in Site 2 was eventually only delivered to trial patients, as opposed to all eligible patients on the ward as planned.

*“for the first half of the trial we did* [*Step 6*] *for all the patients*,* but it was very difficult to catch up with that turnover”.* Site 2 Coordinator.

Available resources were relevant to whether staff prioritised intervention steps, particularly Step 4. Sites took different approaches to organising staff to introduce the toolkit (Step 3). Site 3 felt having only those with heart failure training delivering the toolkit was most appropriate, whereas Site 6 felt non-heart failure specialist staff could implement the intervention, with strong leadership support to increase their confidence.

*“We decided that we didn’t want non-heart failure members of the team explaining…I did extra training myself…how to break the news…for a newly qualified Band 6 pharmacist…it’s quite a big ask.”* Site 3 Pharmacist.

In Site 4, hospital heart failure nurses were also community heart failure nurses. Usual practice as part of this role involved providing existing medicines information to patients in hospital and continuing to speak with patients in community settings. Patients received both this existing information and the toolkit. The nurse was sceptical as to whether the patients were using the toolkit once they returned home.

*“…we see…*[patients] *in the community after discharge and…I’ve yet to have anybody show me their toolkit.”* Site 4 Heart failure specialist nurse.

### Characteristics of individuals

Data were available for ‘knowledge and beliefs about the intervention’ and ‘other personal attributes.’ For example, staff reported some patients felt the intervention was already being undertaken in community pharmacies. Some site coordinators discussed the importance of their ability to increase staff confidence and sense of self-efficacy in delivering MaTI.

In Site 4, staff members found communication with community pharmacies difficult, and in one example overcame this by personally delivering information to the pharmacy.

### Process

The process of implementation was a distinguishing domain, particularly in terms of planning, participant engagement, and executing the intervention.

### Facilitators

#### Planning

There was limited evidence of sites planning for MaTI, however it was a distinguishing concept as some sites appeared to have taken more measures to prepare than others. Site 3 had developed a standard operating procedure to ensure effective implementation of using PharmOutcomes^®^ [15] to transfer information. Training was described as important to prepare staff for MaTI implementation in Site 1 and Site 6.

#### Engaging

Levels of engagement varied across the sites, involving formally appointed internal implementation leaders (site coordinators), champions (specific individuals driving forward implementation), key stakeholders (ward staff), and innovation participants (patients). Key distinctions between sites included lesser engagement of ward staff in Site 1 and Site 2, site coordinator lesser involvement in Site 3, and variation in methods of engaging patients with the toolkit. See additional file 2 for more information on staff implementation roles.

#### Observation data

Site coordinators were engaged in intervention delivery across all sites; however, the level to which they engaged varied. In sites Site 2 and Site 1, the site coordinators had high levels of activity in both delivering the intervention themselves and organising other staff members. Site 3 was an outlier as it was primarily led by pharmacists.

#### Interview data

Educational/career backgrounds and recognising value in the intervention may have influenced site coordinator engagement.

As the site coordinator in Site 6 highlighted;

*“[It’s] really important*,* really good for…* [patients]. *Any education is*,* so they can have self-awareness and ownership of the management of the illness…I can’t see any drawbacks.”* Site 6 Coordinator.

#### Observation data

Pharmacists were champions in Site 2 and Site 3, facilitating implementation. Observations in Site 2 revealed the pharmacy technician taking a pivot role, locating themselves centrally in the ward and coordinating with ward staff to deliver the toolkit, and communicating with patients and staff to identify the community pharmacist.

#### Interview data

In Site 3, the pharmacist led the intervention and described how the site coordinator supported them in doing so. The pharmacist was highly motivated to educate patients, describing their training as a reason for understanding the importance of this. Champions were engaged with steps involving communication with community pharmacy, despite challenges such as duplication of effort.

#### Observation data

Ward staff engagement could be challenging and varied over time. Staff engaged with the toolkit introduction in all but one site where staff engagement deteriorated (Site 1).

#### Interview data

Across most sites, ward staff were less involved in steps related to contact with community pharmacy, Site 1 differed as they were trained in the use of PharmOutcomes^®^ [15] to transfer information to community pharmacy. Training and leadership were important ways of engaging staff, particularly for sites more negative in the compatibility construct. In Site 6 the coordinator described engaging staff through creating a supportive team culture.

*“It was mainly the staff nurses and the Deputy Sister that delivered it…all of the nurses at some point*,* there are probably only a couple of them on the ward that actually didn’t do any delivery of the toolkit…we tried to do it together as a team.”* Site 6 Coordinator.

Task allocation could help engagement. In Site 2 tasks were clearly allocated to different staff members, however this took time to implement. Ward staff lack of engagement led site coordinators to become responsible to deliver more of the intervention than they had capacity for in some sites (Site 2, Site 1).

#### Observation data

Patient engagement was thought to vary according to patients’ individual characteristics, such as number of changes to medicines. However, through structured observations we identified variation in how staff engaged patients.

Full and detailed explanations were provided in some sites. One toolkit introduction involved staff discussing all sections of the toolkit. In over half of cases, we observed patients’ questions being answered by staff. Although some patients had no questions, staff encouraged questions in only one observation. Informing patients of medicines information being sent to the community pharmacist was completed in all but one observation. The MUR or medicines discussion invitation was either not mentioned at all or mentioned very briefly. Emphasising the toolkit’s utility could enhance patient engagement, however some toolkit sections were given greater importance than others.

*“Heart failure specialist nurse…explains the link between pharmacy hospital and GP and how it can break down. Shows toolkit… 4 sections all are important, but 1 and 4 more important, 2 and 3 less so… Meds Checklist and managing meds – says patients really like this section.”* Site 5 Qualitative observation.

We observed the toolkit introduced in stages at some sites (Site 3, Site 4). In Site 3 the patient was gradually introduced to the toolkit, with two staff members before and after lunch, and then it was left with the patient who was encouraged to look at it with a family member. Body language also differed across sites. Some staff members were standing above patients whilst introducing them to the toolkit (Site 1, Site 4) whereas in other cases staff members sat at eye level with the patient (Site 3, Site 5, Site 6).

*“The nurse who first spoke to the patient knelt to be at eye level with the patient*,* smiled and regularly touched the patient’s hand. The patient had trouble seeing and the nurse adjusted the lighting in the room. The pharmacist who gave more detail about the toolkit later engaged with the patient in a similar way*,* pointing out sections of the toolkit.”* (Site 3 Observation).

## Barriers

### Engaging

#### Observation data

Engaging with patients through encouraging questions and listening happened in some cases but less so in others. The pace by which the toolkit was introduced limited patient opportunities for questions in some instances.

*“A little hurried, did not always wait for patient to respond…Ended with asking patient if any questions please ask.”* (Site 4 Observation).

#### Executing

We have used fidelity measures, as outlined in Figs. 2 and 3, to assess the degree to which MaTI was accomplished.

### Implementation across process evaluation sites

The distinguishing CFIR domains above provide insight into why sites implemented the intervention differently, highlighting key barriers and facilitators. An interpretation of these findings for each site is provided below.

### Site 1

Site 1 had one of the highest levels of implementation. Planning for how much staff could achieve without the need for the site coordinator to be constantly present seemed to make a key difference. Moreover, the introduction of the toolkit (step 3) was close to their usual practice which meant that the site coordinator’s presence was not constantly needed. Thus the implementation climate also had an impact as the staff were more receptive to the intervention. Contacting community pharmacists (Step 2b) and Information transfer to community pharmacy (Step 6) were facilitated by ward staff being trained by a pharmacist in the electronic transfer of information using PharmOutcomes^®^. Therefore, ward staff were able to complete this without supervision. However, lack of staff engagement and intervention complexity were distinguishing features in lower levels of completion of the discharge log (Step 4). The reduced engagement in ward staff meant staff coordinators had to be present to drive this aspect of implementation which explains why Step 4 not consistently completed out-of-hours when coordinators were unavailable to support staff.

### Site 2

Site 2 had the lowest levels of completion for contacting the community pharmacist (Step 2b) and toolkit introduction (Step 3). Readiness for implementation particularly had a negative impact on the opportunity to implement the intervention. The site coordinator at site 2 was highly motivated to deliver the intervention and engage staff, with the outer setting characteristic of deeming the intervention as meeting patient needs and resources influencing this. However, ward staff engagement appeared limited. The coordinator described a lack of resource to deliver the intervention as planned, implying a lack of readiness for implementation.

Similar to other sites, intervention characteristics were a barrier as completing the discharge medicines log (Step 4) was seen as duplication of existing practice and therefore the site’s routine discharge summary was used instead of the discharge log (Step 4).

### Site 3

In Site 3 planning and ability were important with standard operating procedures (SOPs) developed, yet it was the lowest implementer for patient told of referral to community pharmacy (Step 5 (63%), information transfer to community pharmacy (step 6) (73%) and check community pharmacist received information (step 7) (6.7%), As with site 1, PharmOutcomes^®^. was used, suggesting that the use of PharmOutcomes^®^ alone was not sufficient to ensure the success of communication with community pharmacy, although information transfer to community pharmacy (Step 6) was completed for the majority of patients. Evidence was limited as to why check community pharmacist received information (Step 7) was not well implemented at site 3, however one key distinction from site 1 (which completed Step 7 well) was that the site coordinator was less involved in site 3. Potentially this additional coordinator input could have supported implementation where the SOP and PharmOutcomes^®^ were not sufficient. Contacting the same community pharmacist following initial contact (Step 2b) was described across sites in general as challenging, and perhaps the site coordinator could have helped to ensure check community pharmacist received information (Step 7) was achieved.

The intervention was pharmacist-driven in Site 3, and the delivery of Step 3 was by pharmacists and nurses trained in heart failure as a specialism. Introduction of the toolkit (Step 3) was highly implemented (93%), and our observations confirmed the quality of delivery of step 3 - it was person-centred and shared effectively between appropriate staff members. Thus, the inner setting, communication, resources, and positive working culture within site 3 had an impact on Step 3 implementation.

### Site 4

Site 4 had higher levels of implementation relative to other process evaluation sites across the seven steps, although *Inform patients they have been referred to their community pharmacist* (Step 5) was slightly less implemented at (67%). The implementation climate (cosmopolitism) was a barrier to implementation. Poor communication with community pharmacy presented a significant challenge, however staff members described making additional efforts of physically visiting the community pharmacies themselves. This may have partly been due to the lack of involvement from hospital pharmacists in this site as highlighted by nursing staff as a barrier to communicating with community pharmacists. Again, as in sites 1 and 2, the site coordinator was the key individual driving the intervention forward.

A nurse reported that patients may have accessed other information sources other than the toolkit post discharge. Thus, the compatibility and relative priority of the intervention seemed to be a barrier to implementation.

### Site 5

In site 5, the intervention was delivered by nurses and a pharmacist. Data from site 5 were limited to observations and surveys. Survey data indicated the intervention was well implemented relative to other process evaluation sites. Our observations confirmed the implementation of step 3. However qualitative observation data revealed that some sections of the toolkit may have been more thoroughly emphasised than other parts.

In one observation, the toolkit introduction primarily focused on the staff members ‘preferred’ sections of the toolkit, with the staff member highlighting to the patient that they disliked the ‘My Medicines’ section of the toolkit.

*“Heart failure specialist nurse…shows toolkit… 4 sections all are important but 1 and 4 more important 2 and 3 less so… Medicines Checklist and managing medicines – says patients really like this section.”* Site 5 Qualitative Observation.

Thus, patient engagement may have been higher on some sections of the intervention than others.

### Site 6

Site 6 had significant challenges contacting community pharmacy in particular, and were the second lowest performing site for contacting the community pharmacist (step 2b), information transfer to community pharmacy (step 6) and check community pharmacist received information (step 7). The implementation climate (cosmopolitism) was a key barrier to this, with identifying and speaking to the correct community pharmacist an additional barrier. The inner setting, readiness for implementation, was a strength with the site coordinator describing the importance of strong leadership for implementation.

### Summary

Intervention complexity was a barrier across sites. Completing the discharge medicines log (step 4) was the least completed step as it was felt to be a duplication of existing documents. Contacting community pharmacy (step 2b), information transfer to community pharmacy (step 6), and check community pharmacy received information (step 7), were a key area of distinction across sites. The implementation climate (cosmopolitism) appeared to be a barrier across sites for these steps. Sites were not well networked with community pharmacy for the purposes of the intervention. The community pharmacy perspective from different sites was unclear as there were limited data from community pharmacy surveys, and community pharmacy interviews were not completed due to the COVID-19 pandemic. However, there were indications overall that community pharmacists were not aware of the intervention, despite being provided with it.

The distinction between sites appears to be their differing approaches to circumventing this barrier. The method of transfer itself may have been a less distinguishing factor than CFIR constructs ‘readiness for implementation’, the amount of resource that sites had, ‘individual characteristics’ of different types of ward staff, and ‘process’ planning through training staff members. For example, Site 4 were relatively successful as they had ward staff physically visiting community pharmacies, site 2 struggled as staff felt there was insufficient resource and therefore became less engaged, and site 3 had less site coordinator presence.

## Discussion

Core MaTI steps were generally delivered as intended across process evaluation sites. Steps that relied only on hospital staff to complete required tasks (Steps 1,2a,3,5 and 6) were completed more frequently than those requiring contacting community pharmacy (Steps 2b, 6 and 7). However, Completion of medicines discharge log (Step 4), was implemented relatively less frequently across sites. Barriers and facilitators to implementation were identified through all CFIR domains and several constructs. However, the most important constructs were intervention complexity, cosmopolitanism (organisation networked), and the available resources organisations had. Hospitals and community pharmacies lacked pre-existing systems of communication which led to difficulties completing steps which relied on this.

The patient toolkit was valued and frequently introduced, with variation in its introduction. However, the medicines discharge log was often not completed, and seen as a duplication of existing systems. Both the log and community pharmacy related steps placed pressure on staff time.

There were distinctions between sites. Some sites were more able to address the challenges associated with communication with community pharmacy as staff were highly motivated, they had effective leadership and resources. However, the higher level of implementation shown by the survey data at some sites such as Site 4 may not be sustainable. High implementers site 4 were physically visiting pharmacies to transfer the information as communication was poor. In Site 1, contact community pharmacist (Step 2b) appeared to be facilitated by site coordinator presence. Thus, how much longer staff would be able to continue such practices is questionable. The CFIR analysis provided an indication of the quality of implementation, in addition to barriers and facilitators to implementation. Our analysis of patient interviews revealed how the MaTI could potentially enable patients to increase medicines knowledge, be alerted to seek help, communicate more effectively with health care professionals, provide information, support existing care, and support systems and be reassured when professional support was unavailable. However, patients were less able to benefit from these enhancements where they faced issues with design and delivery of the toolkit, as well as sources of support available to them within the community [10]. The toolkit delivery was inconsistent, with some staff members missing sections, and not always taking time to effectively engage with the patient. The Capability, Opportunity and Motivation COM-B model suggests that for someone to engage in a behaviour they must be physically and psychologically capable to use opportunities through motivators [23]. Staff may be lacking opportunity to engage patients through lack of resource. Moreover, ward pharmacists may have had more capability and opportunity to communicate with community pharmacists where communication systems were already in place. Effective collaboration between hospital and community pharmacists can help to establish continuity of care.

### Implications for policy and practice

Whilst the analysis did indicate the benefit of personalising the toolkit according to patient need, greater guidance around the toolkit introduction may be necessary when implementing the intervention, particularly on a wider scale. We know from patient interviews that the quality of delivery makes a significant difference to patient experiences, impacting on whether patients feel the toolkit is important to engage with, or what the role of community pharmacy is [10].

From an implementation perspective, the lack of receptiveness from community pharmacy led to hospital staff investing more resources to refer patients and send discharge information. Thus, sustainable implementation relies on developing greater engagement with community pharmacy. When the intervention was designed, community pharmacy involvement was considered. We wished to understand how the intervention could work in a real world setting within existing commissioned services. Moreover, it was necessary to ensure control sites were not contaminated by exposing community pharmacies to the intervention, which may have theoretically activated them to intervene. Therefore, it was decided that community pharmacies first contact would be by the hospital with a cover letter at the point of discharge. We could not therefore work with community pharmacies to resolve usages of different electronic systems. For the intervention to be transferable across settings, existing practices and systems across primary and secondary care need to be considered [24–27]. Despite steps being carried out in Site 4, positive impacts of implementation may have been reduced because of the competing information providing by the heart failure specialist nurse who was a community heart failure nurse. It may potentially be difficult to implement ISCOMAT in other systems in Europe. However, there is important learning from our innovative practice which may be learnt from or applied.

### Strengths and limitations

Our strengths include using a mixed methods approach. Multiple key data sources were collated before the impact of the COVID-19 pandemic. The toolkit introduction (Step 3), a vital step of the intervention, was explored fully, revealing the importance of ‘process’ for the MaTI’s implementation, as the hospital observations provided rich data. However, a structured observation was not completed in Site 2, as it was not possible to know in advance when the toolkit would be introduced to patients, and due to geographical distance, it was not possible for researchers to respond quickly. We were able to conduct most hospital staff interviews despite the COVID-19 pandemic, which were crucial in helping us to identify and explore barriers and facilitators to implementation. Protocol changes due to the COVID-19 pandemic included the reduction in hospital staff interviews from 30 to 11, 2 community pharmacy interviews where 10 were planned, 4 process evaluation community pharmacy surveys, and no community heart failure nurse surveys collated where a maximum of 30 were planned. The limited number of process evaluation community pharmacy interviews and surveys were not analysed. Only the evaluation stage was affected by the COVID-19 pandemic. Implementation of the intervention at process evaluation sites ended prior to the pandemic. All observation of implementation and patient interviews were conducted prior to the pandemic. Staff interviews were completed prior to and during the pandemic about events prior to the pandemic.

Trial survey data from community pharmacies for process evaluation sites were collated prior to the pandemic. Community pharmacy survey data from non-process evaluation sites were collected both prior to the pandemic and during the pandemic. It was a study limitation that community pharmacies could not have been given more notice of their potential role in the post discharge process with medication reconciliation and MUR. However, we wished to understand how the intervention could work in a real world setting and ensure control sites were not contaminated. It was also a study limitation that data were not collated about any differences in the types of community pharmacy, which could potentially exist.

Our study was limited to six process evaluation sites which reduces generalisability. However, we were able to recruit a diverse sample of sites based on key characteristics that could influence implementation including a range of university and non-university hospitals, differing methods for transferring medicines discharge information to community pharmacists and covering different geographic areas of England. Moreover, fidelity data were collected for all intervention sites.

Applying an inductive approach to analysis risked confirmation bias. One of the researchers involved in the process evaluation (HI) had also been heavily involved in implementing the intervention. However, the results were agreed by both CP and HI and confirmed with the wider research team. We planned to utilise the CFIR purely for analysis purposes, which is common practice across implementation studies. However, we acknowledge that the process may have been enhanced by applying the CFIR more extensively during earlier study phases, such as assessing sites pre-implementation or employing a scoring system as advocated by Damschroder et al. (2013) had there been more extensive data. Our pre-Covid-19 plan had been to collect survey data from hospital staff, community pharmacists and heart failure nurses, as well as interviews with community pharmacists and site 5 staff, but this was not possible. Consequently, the analysis was based on less data than originally planned. Moreover, unfortunately we were not able to observe specific policies or organisational directives that made discharge easier at one site than another. Thus, some domains may have been more or less distinguishing.

## Conclusion

The MaTI was relatively well implemented with hospital staff completing intervention steps. However, steps involving community pharmacy were more challenging to implement. Key facilitators included staff motivation to deliver the intervention when it was felt to meet patients’ needs. Patient education was regarded as important for supporting patients to continue monitoring and understanding their medicines. Barriers were identified including the interventions complexity, how well hospitals were networked with community pharmacies and availability of hospital resources.

The findings from this study will support an explanation of the main trial findings when available. Community pharmacy and hospitals need more effective systems of communication to allow safe transfer of medicines information at transitions of care; thus, system level issues need to be addressed to support the wider implementation if the MaTI is shown to be effective.

## Supplementary Information

Additional file 1.

Additional file 2.

Additional file 3.

Additional file 4.

Additional file 5.

Additional file 6.

Additional file 7.

## Data Availability

All data requests should be submitted to the corresponding author for consideration and would be subject to review by a subgroup of the team. Access to anonymised data may be granted following this review. All data-sharing activities would require a data-sharing agreement.

## Abbreviations

MaTI: Medicines at Transition Intervention
CFIR: Consolidated Framework for Implementation Research
MUR: Medicines Use Review
