# Supplementary material for: Implementing a Medicines at Transitions Intervention (MaTI) for patients with heart failure: a process evaluation of the Improving the Safety and Continuity Of Medicines management at Transitions of care (ISCOMAT) cluster randomised controlled trial
Source: BMC Health Serv Res. 2024 Oct 9;24:1210. doi: 10.1186/s12913-024-11487-x (PMC11465536; doi:10.1186/s12913-024-11487-x)
Supplement: Supplementary file 1 — Additional file 1. [file 12913_2024_11487_MOESM1_ESM.pdf]

Additional file 1: Consolidated criteria for reporting qualitative studies COREQ 32-ITEM CHECKLIST

Tong A, Sainsbury P, Craig J. (2007) Consolidated criteria for reporting qualitative research (COREQ): a 32- item checklist for interviews and focus groups. International Journal for Quality in Healthcare: 19:349 – 357

No. item	Guide questions/description	Page no.
Domain 1: Research team and reflexivity		
1. Interviewer/facilitator	Which author/s conducted the interview?	3
2. Credentials	What were the researcher's credentials?	3
3. Occupation	What was their occupation at the time of the study?	3
4. Gender	Was the researcher male or female?	3
5. Experience and training	What experience or training did the researcher have?	3
6. Relationship with participants established	Was a relationship established prior to study commencement?	3
7. Participant knowledge of the interviewer	What did the participants know about the researcher?	3
8. Interviewer characteristics	What characteristics were reported about the interviewer/facilitator?	3
Domain 2: Study design		
9. Methodological orientation and Theory	What methodological orientation was stated to underpin the study?	2
10. Sampling	How were participants selected?	3
11. Method of approach	How were participants approached?	3
12. Sample size	How many participants were in the study?	3-4
13. Non-participation	How many people refused to participate or dropped out? Reasons?	4
14. Setting of data collection	Where was the data collected?	3
15. Presence of nonparticipants	Was anyone else present besides the participants and researchers?	3
16. Description of sample	What are the important characteristics of the sample?	4
17. Interview guide	Were questions, prompts, guides provided by the authors?	Additional file 4 and 6
18. Repeat interviews	Were repeat interviews carried out?	n/a
19. Audio/visual recording	Did the research use audio or visual recording to collect the data?	4
20. Field notes	Were field notes made during and/or after the interview?	n/a
21. Duration	What was the duration of the interviews	4
22. Data saturation	Was data saturation discussed?	n/a
23. Transcripts returned	Were transcripts returned to participants for comment and/or correction?	n/a
Domain 3: Analysis and findings		
24. Number of data coders	How many data coders coded the data?	4
25. Description of the coding tree	Did authors provide a description of the coding tree?	4

26. Derivation of themes	Were themes identified in advance or derived from the data?	4
27. Software	What software, if applicable, was used to manage the data?	4
28. Participant checking	Did participants provide feedback on the findings?	n/a
29. Quotations presented	Were participant quotations presented to illustrate the themes/findings? Was each quotation identified?	5-12
30. Data and findings consistent	Was there consistency between the data presented and the findings?	5-12
31. Clarity of major themes	Were major themes clearly presented in the findings?	5-12
32. Clarity of minor themes	Is there a description of diverse cases or discussion of minor themes?	5-12
