# Supplementary material for: Implementing a Medicines at Transitions Intervention (MaTI) for patients with heart failure: a process evaluation of the Improving the Safety and Continuity Of Medicines management at Transitions of care (ISCOMAT) cluster randomised controlled trial
Source: BMC Health Serv Res. 2024 Oct 9;24:1210. doi: 10.1186/s12913-024-11487-x (PMC11465536; doi:10.1186/s12913-024-11487-x)
Supplement: Supplementary file 2 — Additional file 2. [file 12913_2024_11487_MOESM2_ESM.pdf]

Additional file 2: Characteristics of process evaluation sites

Site ID	No. of beds	Type of ward	Method of transferring information to community pharmacy during intervention implementation	No. trial patients recruited	Staff delivering MaTI (from observation and interview data) and activities undertaken (from interview data)
Site 1	25	Cardiology	IT system PharmOutcomes® for intervention use only	65	*Staff delivering:* Heart failure specialist nurse, research nurse, ward nurses, pharmacist (observation data).<br>*Activities:* Site coordinator consenting patients to trial, training staff in step 3 at beginning, identified and liaised with community pharmacy and GPS, discharged MaTI patients. Trained staff, step 6 by staff nurse or pharmacist (interview data).
Site 2	29	Cardiology /ACU	Mail by site coordinator PharmOutcomes® access not granted	73	*Staff delivering:* Heart failure specialist nurse, research nurse, ward nurses, pharmacy technician (observation data), ward clerk, doctors (interview data)<br>*Activities:* Pharmacy technician identify community pharmacists, for trial patients only later in implementation. Doctors encouraging staff. Nurses implementing step 3. Site coordinator conducted research and most implementation activities, encouraging staff, transfer of discharge letters. Nurses step 3 (Interview data).
Site 3	29	Cardiology /CCU	PharmOutcomes® by ward pharmacist	39	*Staff delivering:* Heart failure specialist nurse, research nurse, ward nurses, pharmacist (interview and observation data).<br>*Activities:* Research nurses and pharmacist identify patients. Pharmacist step 3, created ISCOMAT SOP. Using PharmOutcomes® to send information to community pharmacy (Interview data)
Site 4	19	Cardiology	Email/hand delivered by site coordinator and ward staff<br>Asked community pharmacists for preference	49	*Staff delivering:* Heart failure specialist nurse, research nurse, ward nurses, pharmacist (observation data). Site coordinator (interview data)<br>*Activities:* 4 research nurses recruited, doctors notified patients, ward staff delivering toolkit, site coordinator identify patients and step 6. Heart failure specialist nurse also has community and research role.
Site 5	17	Cardiology /CCU	Mail by site coordinator	21	*Staff delivering:* Heart failure specialist nurse, research nurse, ward nurses, pharmacist (observation data). *Activities:* Limited data due to no interviews. Ward nurses introducing step 3 (observation data).

Site 6	12	Cardiology	Mail by site coordinator	19	*Staff delivering:* Heart failure specialist nurse, ward nurse, ward sister, deputy sister, pharmacist (observation data).<br>*Activities:* Step 3 by nurses. Site coordinator over seeing training and identifying patients (interview data).
