# Supplementary material for: Implementing a Medicines at Transitions Intervention (MaTI) for patients with heart failure: a process evaluation of the Improving the Safety and Continuity Of Medicines management at Transitions of care (ISCOMAT) cluster randomised controlled trial
Source: BMC Health Serv Res. 2024 Oct 9;24:1210. doi: 10.1186/s12913-024-11487-x (PMC11465536; doi:10.1186/s12913-024-11487-x)
Supplement: Supplementary file 3 — Additional file 3. [file 12913_2024_11487_MOESM3_ESM.pdf]

Additional file 3: ISCOMAT Structured Observation Schedule

**Site:**	**Date:**
**Observer:**	**Time started:**
☐ **Patient agreed to observation?**	**Time completed:**
☐ **Staff member agreed to observation?**	

Note: Focus on my medicines toolkit questions 1-18. Answer 19- if possible.

Department/Ward details

**Setting of the observation** *(Provide general observations of department/ward below)*	
Number of Beds on Ward	
Number of Patients on Ward	
Number of Staff on Ward	
Ward layout	

Type of Staff on Ward	Number
Sister	
Staff Nurse	
Health Care Assistant	
Junior Doctors	
Pharmacist	
Consultant	
Other	

Observation notes

**Details of Health Care Professional being Observed**	
Job Title & Unique Identifier	
Qualifications	
Usual place/area of work e.g. ward	
Length of time working in that role/length of time qualified	

	**Discussion with toolkit**	**Criteria Met**			**Comments**<br>Include explanation if the answer is "part" and for additional observations
**Overall**					
**1**	Was the MATI toolkit introduced during discussion with the patient?	**Yes**	**No**	**Part**	*How long did the staff spend with the patient explaining the toolkit?*

**2**	Did the discussion include all the sections of the toolkit?	**Yes**	**No**	**Part**	
**3**	Were questions posed by the patients answered by staff?	**Yes**	**No**	**Part**	
**4**	Were patients encouraged to ask questions?	**Yes**	**No**	**Part**	
**MATI Section 1: My healthcare team**					
**5**	Did the staff member help the patient think about which healthcare professionals are in their team?	**Yes**	**No**	**Part**	

**6**	Did the staff members help the patient understand how they can contact each healthcare professional in their team?	**Yes**	**No**	**Part**	
**7**	Did the staff member highlight that the patient’s community pharmacy will be informed about their discharge and sent the patients discharge medicines information and advice note?	**Yes**	**No**	**Part**	
**8**	Was it made clear to the patient that the community pharmacy will invite the patient for a Medicines Use Review (MUR)/New Medicines Service/DRUM soon after being discharged?	**Yes**	**No**	**Part**	*Which type of review was mentioned?*
**9**	Did the staff member inform the patient that if they have not heard anything from the pharmacy for 2	**Yes**	**No**	**Part**	

	weeks they should contract them to ask for a MUR/DRUM/conversation with their pharmacist?				
**MATI Section 2: My medicines checklist**					
**10**	Was the medicines checklist explained to the patient?	**Yes**	**No**	**Part**	
**11**	Did the staff member explain how each item in the checklist should be ticked?	**Yes**	**No**	**Part**	
**12**	Did the staff member ensure that the patient understood that if there is an item they are unable to tick they should consult a member of their healthcare team?	**Yes**	**No**	**Part**	

**13**	Was the patient shown the space for patients to write in their future healthcare appointments?	**Yes**	**No**	**Part**	
**MATI Section 3: Managing my medicines**					
**14**	Was the patient shown the descriptions of medicines?	**Yes**	**No**	**Part**	
**15**	Were the patients shown the description of each medication?	**Yes**	**No**	**Part**	
**16**	Was the patient advised to read section 3 in hospital and when they are at home?	**Yes**	**No**	**Part**	

**MATI Section 4: Managing my symptoms**					
**17**	Did the staff member explain the traffic light guide?	**Yes**	**No**	**Part**	
**18**	Did the staff member point out the symptom log that patients can complete?	**Yes**	**No**	**Part**	
**Additional observations**					
**19**	Did the staff member complete the MaTI checklist MaTI Form A?	**Yes**	**No**	**Part**	

**20**	Was MaTI form B completed?	**Yes**	**No**	**Part**	
**21**	Did the staff member check that it is was OK to transfer discharge information to the community pharmacy?	**Yes**	**No**	**Part**	
**22**	Were patients helped to identify a pharmacy if needed?	**Yes**	**No**	**Part**	
**23**	Was the fold out discharge log completed?	**Yes**	**No**	**Part**	

**24**	Was the discharge advice note communicated through the MaTI Community Pharmacy Cover Letter?	**Yes**	**No**	**Part**	
**25**	Did the staff member make contact with community pharmacy by phone to check the discharge information has been received?	**Yes**	**No**	**Part**	
