# Supplementary material for: Implementing a Medicines at Transitions Intervention (MaTI) for patients with heart failure: a process evaluation of the Improving the Safety and Continuity Of Medicines management at Transitions of care (ISCOMAT) cluster randomised controlled trial
Source: BMC Health Serv Res. 2024 Oct 9;24:1210. doi: 10.1186/s12913-024-11487-x (PMC11465536; doi:10.1186/s12913-024-11487-x)
Supplement: Supplementary file 4 — Additional file 4. [file 12913_2024_11487_MOESM4_ESM.pdf]

Additional file 4: ISCOMAT Unstructured Observation Fieldwork Notes

**Site:**	**Date:**
**Observer:**	**Time started:**
**Patients agreed to observation?** ☐	**Time completed:**
**Staff members agreed to observation?** ☐	

Note: Prior to observations consider the consolidation framework e.g. the interventions characteristics, outer setting, inner setting, characteristics of individuals and process

Prompts

How easy is it for staff to follow the introduction of the toolkit exactly as intended? If not, how and why is it not?

How useable is the toolkit?

Do staff members receive any praise for using the toolkit?

What systems to staff use to communicate with one another e.g. one computer, tablets, meetings?

Are sensitive topics discussed in private settings?

Are staff members receptive to the intervention?

What reasons do staff members provide for not introducing the toolkit to patients?

What comments do staff members make about the value of MATI? E.g. ‘we do it anyway’, ‘straight forward’, ‘hard to follow’?

What comments do staff members make about their own ability to complete MATI?

How is MATI discussed with other staff members?

Interruptions?

Barriers?

Please write notes below:
