# Supplementary material for: Implementing a Medicines at Transitions Intervention (MaTI) for patients with heart failure: a process evaluation of the Improving the Safety and Continuity Of Medicines management at Transitions of care (ISCOMAT) cluster randomised controlled trial
Source: BMC Health Serv Res. 2024 Oct 9;24:1210. doi: 10.1186/s12913-024-11487-x (PMC11465536; doi:10.1186/s12913-024-11487-x)
Supplement: Supplementary file 5 — Additional file 5. [file 12913_2024_11487_MOESM5_ESM.pdf]

## Additional file 5: Community pharmacy survey

ISRCTN66212970

**ISCOMAT** Managing Medicines for Heart Failure Patients

**FORM 29** Page 1 of 1

# Community Pharmacy Data

| Participant Initials | | Date of Birth | Day Month Year | Participant ID | Site Code | Trial No |
|---|---|---|---|---|---|---|

**Instructions:**

- You have been identified as the nominated pharmacist for the above patient
- The patient is taking part in the ISCOMAT trial and has provide consent for their data to be collected from their nominated pharmacist
- Please complete this form and return to the CTRU in the envelope provided
- If you require additional information on completion of this form, please contact the ISCOMAT trial team: **iscomat@leeds.ac.uk**; tel: **0113 343 7588**
- Please don't return the accompanying letter, as this contains the full name of the patient

1. I received a copy of the patient's discharge advice note with the patient's medicines list ☐ Yes ☐ No

   **If yes**, on what date did you receive this? Day Month Year

2. I reconciled the patient's medicines ☐ Yes ☐ No

3. The patient has been invited for a post-discharge Medicines Use Review (MUR) or a discussion about their medicines ☐ Yes ☐ No

4. A Medicines Use Review (MUR) / medicines discussion was completed for the patient

   ☐ Yes – MUR ☐ Yes – medicines discussion ☐ No

   **If yes (either MUR or medicines discussion)**, on what date did this take place? Day Month Year

   **If no**, please state why not

5. Did you undertake the voluntary ISCOMAT/MaTI CPPE learning package? ☐ Yes ☐ No

6. Did anyone else in your pharmacy undertake the CPPE learning package?

   ☐ Yes ☐ No ☐ Unknown

   **If yes**, was it:

   ☐ A pharmacist
   ☐ A dispensing/pharmacy assistant
   ☐ A medicines counter assistant
   ☐ Other, please specify

Name of pharmacist

Signature of pharmacist Date Day Month Year

Name of pharmacy

| For office use only | Computerised Date Initials | Verified/Checked Date Initials |
|---|---|---|

Last Page ■

Version 2.0 21/08/2018
