# Supplementary material for: Implementing a Medicines at Transitions Intervention (MaTI) for patients with heart failure: a process evaluation of the Improving the Safety and Continuity Of Medicines management at Transitions of care (ISCOMAT) cluster randomised controlled trial
Source: BMC Health Serv Res. 2024 Oct 9;24:1210. doi: 10.1186/s12913-024-11487-x (PMC11465536; doi:10.1186/s12913-024-11487-x)
Supplement: Supplementary file 6 — Additional file 6. [file 12913_2024_11487_MOESM6_ESM.pdf]

Additional file 6: Hospital staff interview schedule

1. Please tell me about your experience working in the hospital.

Prompt:

- How long have you been working at the hospital?

2. Please tell me about your experience working on the ward.

Prompts:

- How long have you been working on the ward and hospital?
- What is the patient population on the ward? And in your experience how has it changed? (Older people, types of condition-heart failure)
- Have you ever been or are you involved in research here?

3. Tell me about your role in the Medicines At Transitions Intervention (MaTI) (ISCOMAT)

Prompts:

- Which parts of the intervention (show 7 steps as reminder) were you responsible for delivering? Did this change over time?
- Did you get used to delivering MaTI, did it become a habit?
- Were you prompting staff?

If applicable- Tell us about how you identify patients for ISCOMAT?

Prompts:

- Possible patients on other wards?
- Screening patient admissions lists?
- Consulting with heart failure specialist nurse/other colleagues?

4. Has your involvement in MaTI changed from your usual role or not?

Prompt:

- How?

5. Tell me about the other staff involved in delivering the Medicines At Transitions Intervention (MaTI). (ISCOMAT)

Prompts:

- What are their roles (team – doctors, nurses, hospital pharmacy / others?)
- How do you work with each other to deliver the intervention for these patients?
- How would you improve how you work together?
- What problems, if any, do you experience in delivering the intervention?

6. Has a heart failure specialist nurse been involved?

7. Did you get involved in identifying patients' community pharmacies?

Prompts:

- How easy or difficult was it to identify a patient's community pharmacy?
- How much help did they need?
- Did you contact the community pharmacy to tell them about the patient?
- Why?
- Why not?

8. Did you personalise the toolkit for patients to use? (Show them a copy)

Prompts:

- Write in medicines lists?
- Write in names of healthcare practitioners?
- How long did this take?

9. How much time did you spend explaining the Medicines Toolkit to them?

Prompts:

- Did you leave the toolkit with the patients or go through it with them?
- How many times did you talk to them about it?
- Could you tell me how you introduced it to them?

- What did you tell them about each section? (The traffic lights? The symptoms journal? The medicines list? The healthcare team section?)
- Did you go through the healthcare appointments section?
- What sort of questions did patients have about the Medicines Toolkit?
- Were you able to answer those questions?
- What additional information did you need to answer patients' questions?
- How easy or difficult was it to find that information?
- How involved were patients / family members / carers?

10. In what ways, if any, did you provide ongoing support to patients in their use of the Medicines Toolkit during their hospital stay?

Prompts:

- Did you help them complete the 'my healthcare team' section?
- How did you help?
- Did you talk to patients in any detail about their medicines using the toolkit?
- Did you complete the list of patients' medicines?

11. Did you discharge any ISCOMAT patients and if so did you use the toolkit?

Prompts:

- Were you involved in ISCOMAT patients discharge?
- Completing discharge medicines information and titration plans?
- Going through the my health care team with patients?
- Discussing the symptom traffic lights?
- What sort of questions, if any, did patients ask? How involved were the patients? And carers / family members?

12. How much time did you have to deliver the MATI?

Prompts:

- How did that impact on how well you were able to use it?
- Was MATI integrated into usual practice?

13. Were you the staff member who sent discharge medicines information to the patient's community pharmacy?

- [If yes] how did you do this?

Prompts:

How did you contact the pharmacy?

How easy was it to do this?

Did you receive any queries from the community pharmacy?

- [If no] how involved were you in this process?

Prompts:

Who compiled this information?

Did they ask for your help?

14. Have working relationships between staff changed since MaTI was introduced?

Prompts:

- How?
- Do you feel you can approach other staff members to ask them to deliver aspects of the intervention? (Site coordinator)

15. How prepared did you feel to deliver the MATI? (ISCOMAT)

Prompts:

- Did you take the ISCOMAT e-learning module? If yes, how useful did you find this training? Why?
- What in particular did you learn from the module?
- What, if anything, did you learn about the ISCOMAT intervention? How confident did you feel delivering the MATI? How could you have been more confident?
- How often did you refer to the ISCOMAT intervention guide?

16. How about the on-site training? How well did it prepare you for delivering the MaTI?

Prompts:

- Did you attend the face-to-face training provided by the research team?
- If not who trained you?
- Has the training been effectively shared between staff members?
- How much were you able to practice different parts of delivering it during the training?
- How much were you able to resolve problems you might encounter delivering the MaTI during the training?
- Were you given any documents to help you deliver MaTI? (whether provided by the study team or not)
- How useful were the documents you received to help you deliver MaTI?
- The site co-coordinators’ guide or the seven steps?
- How about the materials to deliver the MaTI?
- The checklist?
- The pharmacy referral forms?
- The community pharmacy record form?

Implementation of MaTI at this site

17. What have been the main challenges for this site in delivering the steps?

Prompts:

Step 1 – Identify MaTI patients and begin/complete the MaTI checklist

Step 2 – Identify patients’ community pharmacist and contact the pharmacist to let them know the patient is in hospital and discharge information will be sent

Step 3 – Introduce/ talk through patients to the ‘My Medicines Toolkit’ booklet

Step 4 – Complete the foldout discharge heart failure medicines log

Step 5 – Tell the patient you will be referring them to their community pharmacy for a follow up and that they will be invited to a medicines use review/discussion about medicines

Step 6 – Transfer the patients discharge advice letter and medicines list to the community pharmacy

Step 7 – Check that the information has been received by the community pharmacy

18. What has facilitated the use of these steps in this hospital?

Prompts:

[go through steps]

19. [Give an explanation of the distinction between implementation and trial, are they aware?] Has the intervention been implemented at ward level or to trial participants?

Overall

20. Overall, how important do you think it is to deliver the MATI for heart failure patients?

Scaling up

21. If we were to roll out this intervention on a wider scale to other patients with different conditions or in different care settings, how do you think we could do this?

Prompts:

- What might we have to change/adapt?
- What you think the barriers and facilitators might be?
- How could we overcome the challenges?

22. Has being part of the MaTI study changed/influenced the way that you deliver care on the ward?

Prompts:

- More focus on heart failure patients' education?
- Longer discussion about meds?
- Modified information/materials given to patients?

Do you have any other comments about the ISCOMAT Medicines At Transitions Intervention?

Thanks and debrief
