# Supplementary figures and images for: Implementing a Medicines at Transitions Intervention (MaTI) for patients with heart failure: a process evaluation of the Improving the Safety and Continuity Of Medicines management at Transitions of care (ISCOMAT) cluster randomised controlled trial
Source: BMC Health Serv Res. 2024 Oct 9;24:1210. doi: 10.1186/s12913-024-11487-x (PMC11465536; doi:10.1186/s12913-024-11487-x)

## Additional file 7: Structured observations

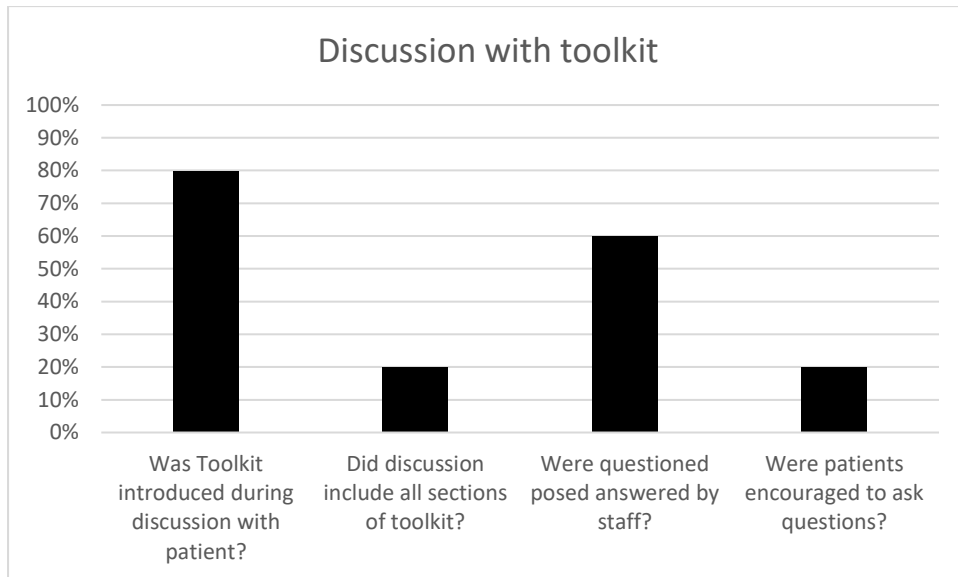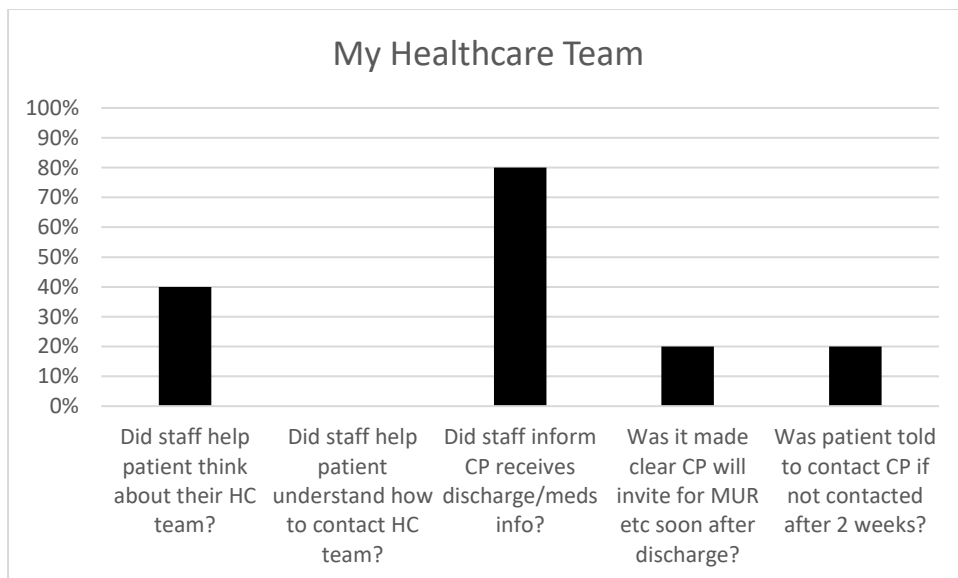

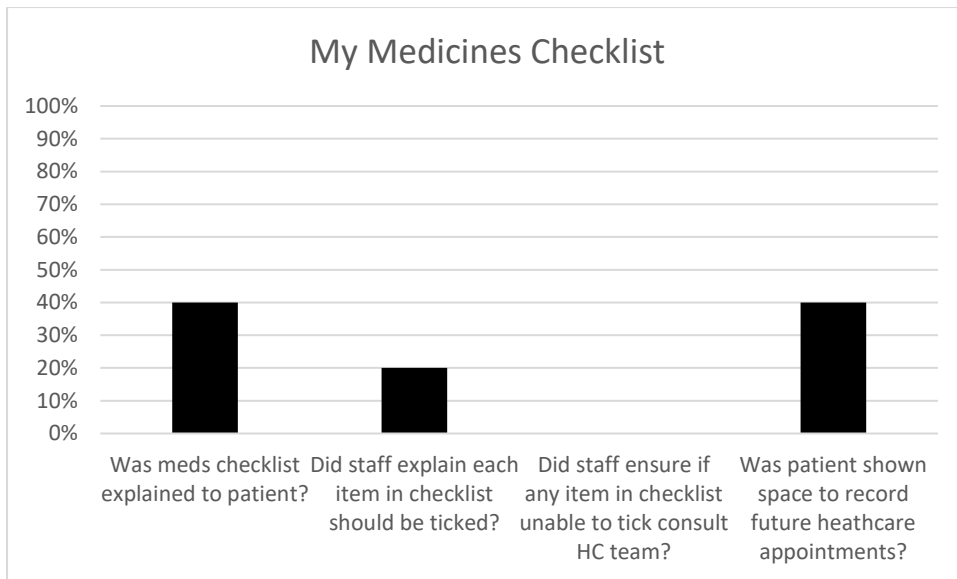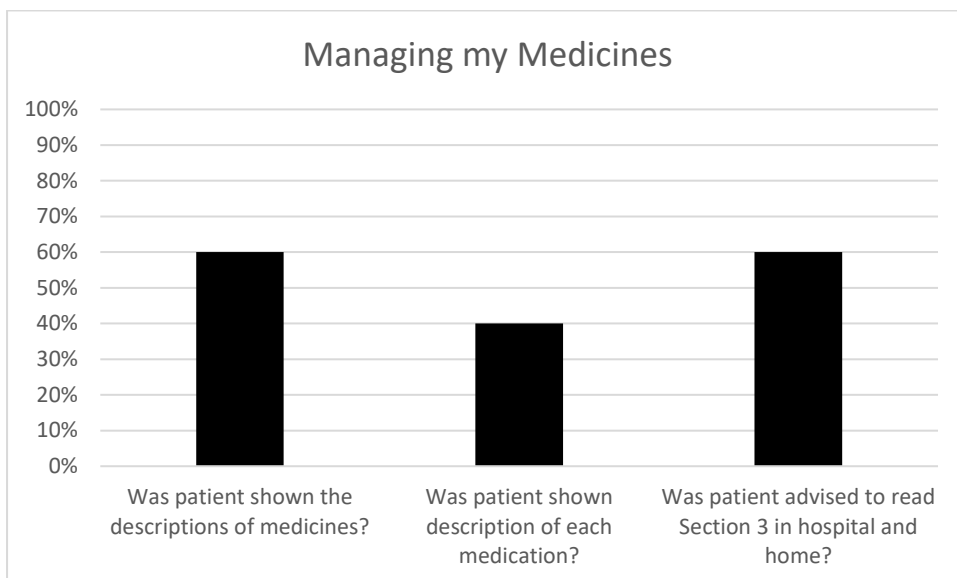

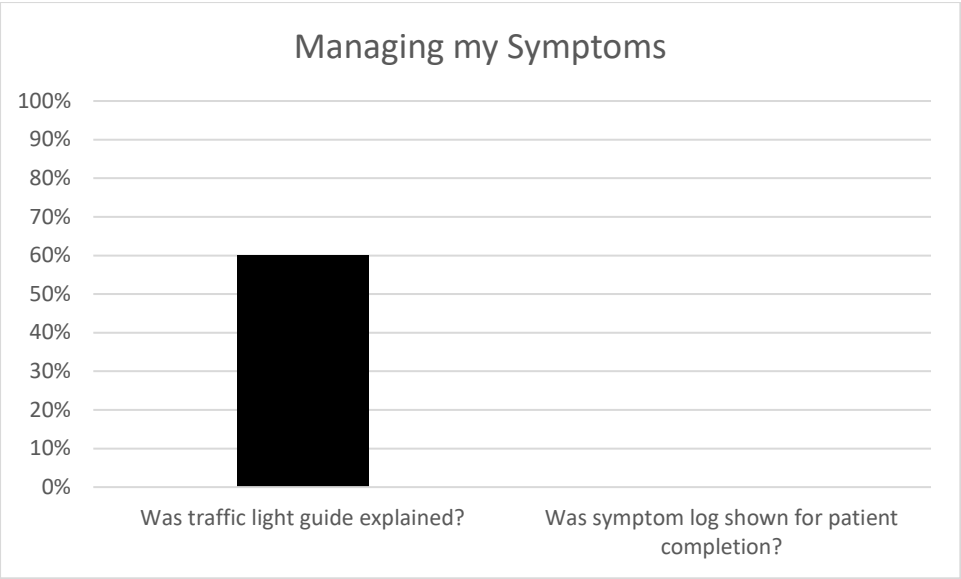

Supplement: Supplementary file 7 — Additional file 7. [file 12913_2024_11487_MOESM7_ESM.pdf]
